# Pakistan’s path to universal health coverage: national and regional insights

**DOI:** 10.1186/s12939-024-02232-1

**Published:** 2024-08-15

**Authors:** Di Yang, Zlatko Nikoloski, Ghazna Khalid, Elias Mossialos

**Affiliations:** 1 LSE Health, The London School of Economics and Political Science, Houghton Street, London, WC2A 2AE UK; 2 Health Services Academy, Park Road, Chak Shahzad, Islamabad, 44000 Pakistan

**Keywords:** Universal health coverage index, Pakistan, Subnational analysis, Catastrophic health expenditure

## Abstract

**Background:**

Universal Health Coverage (UHC) is a common health policy objective outlined in the Sustainable Development Goals. With provincial governments taking the initiative, Pakistan has implemented and extended UHC program amid a complex public health landscape. In this context, we assess Pakistan’s progress toward achieving UHC at the national and subnational level.

**Methods:**

We use data from the Demographic and Health Surveys and the Household Integrated Economic Survey to construct a UHC index at the national and subnational level for 2007, 2013, and 2018. Furthermore, we use Concentration Index (CI) and CI decomposition methodologies to assess the primary drivers of inequality in accessing medical services. Logistic regression and Sartori’s two-step model are applied to examine the key determinants of catastrophic health expenditure (CHE).

**Results:**

Our analysis underscores Pakistan’s steady progress toward UHC, while revealing significant provincial disparities in UHC progress. Provinces with lower poverty rate achieve higher UHC index, which highlights the synergy of poverty alleviation and UHC expansion. Among the examined indicators, child immunization remains a key weakness that one third of the children are not fully vaccinated and one sixth of these not-fully-vaccinated children have never received any vaccination. Socioeconomic status emerges as a main contributor to disparities in accessing medical services, albeit with a declining trend over time. Household socioeconomic status is negatively correlated with CHE incidence, indicating that wealthier households are less susceptible to CHE. For individuals experiencing CHE, medicine expenditure takes the highest share of their health spending, registering a staggering 70% in 2018.

**Conclusion:**

Pakistan’s progress toward UHC aligns closely with its economic development trajectory and policy efforts in expanding UHC program. However, economic underdevelopment and provincial disparities persist as significant hurdles on Pakistan’s journey toward UHC. We suggest continued efforts in UHC program expansion with a focus on policy consistency and fiscal support, combined with targeted interventions to alleviate poverty in the underdeveloped provinces.

**Supplementary Information:**

The online version contains supplementary material available at 10.1186/s12939-024-02232-1.

## Background

Enacted in 2015, Sustainable Development Goals (SDGs) set the agenda to achieve a range of human development targets by 2030. Among these goals, universal health coverage (UHC) stands as a critical indicator, highlighting the global commitment to ensuring equitable access to essential health services for all individuals, regardless of their socioeconomic status or geographic location [1]. Researchers have been tracking countries’ progress toward UHC, particularly for low- and middle-income countries (LIMCs) [2–5]. Pakistan’s experience in delivering essential health services and progressing toward UHC can offer valuable insights to the Global South, especially for other LMICs with limited medical resources and low institutional development.

Pakistan, with a per capita GDP of $1596.7 in 2022, falls in the group of lower-middle income economies by the World Bank classification [6, 7]. A population of 236 million makes Pakistan the fifth most populous country in the world [7]. Pakistan has been lagging in key health care indicators. Infant mortality rate was 53 deaths per 1,000 live births in 2021, which was the 16th highest in the world according to the World Bank [7]. Although maternal mortality ratio (MMR) has been steadily declining, Pakistan’s MMR is still substantially higher than the neighboring countries of India and Bangladesh. Pakistan reported 154 deaths per 100,000 live births in 2020, compared to 103 in India and 123 in Bangladesh [7]. Infectious diseases such as polio and malaria remain endemic in Pakistan, which aggravates the burden on the health care system [8, 9]. Chronic understaffing in the health care system is a key barrier on Pakistan’s path toward UHC. Pakistan reported only 1.1 physicians per 1,000 population in 2019, substantially lower than the world average of 1.7 physicians per 1,000 population [7]. Meanwhile, the existing literature suggests a minimum of two physicians per 1,000 population to achieve UHC [10]. Furthermore, the government’s persistent underinvestment in health care stalls Pakistan’s progress toward UHC. The existing literature suggests 5% as the benchmark for general government health spending as percentage of GDP [11, 12]. However, this indicator has been stagnant at 1% for Pakistan since 2000 [13]. Meanwhile, domestic private health expenditure consistently accounts for more than half of current health expenditure with a peak of 80% in 2006 [13].

Against this challenging public health background, Pakistan has implemented several programs to improve maternal care and women’s health with the help from various international organizations, such as the World Bank and the Asian Development Bank [14, 15]. The Lady Health Worker Program (LHWP), launched in 1993, extends basic health services to remote and marginalized areas, while bridging the gap between local communities and health care providers [16]. The fourth external evaluation of the program highlights the impact of lady health workers, particularly in maternal and antenatal care: households covered by the program are more likely to use modern contraception method and children are more likely to achieve full immunization [17]. Moreover, Pakistan has taken UHC-inspired reforms and gradually rolled out nationwide health care coverage. First introduced in Khyber Pakhtunkhwa (KP) province in 2015 and fully subsidized by the government, “Sehat Sahulat Program” extends health care coverage to families below the poverty line for treatment up to one million Pakistani Rupees (PKR) per family per year, which is equivalent to $31,776 in purchasing power parity (PPP) [7, 18]. Expanded in late 2020, the program now covers all residents in the KP province [19]. Similar UHC programs are subsequently rolled out to other provinces in the country and gradually push Pakistan toward the UHC target [18]. Pakistan’s bottom-up approach to rolling out UHC coverage can be characterized as provincial government taking the initiative and gradually replicating to other provinces, which is in contrast to other countries’ top-down approach where the central government taking the lead to formulate a national UHC program.

Pakistan’s UHC efforts are in the background of nationwide poverty reduction over decades with innovative poverty alleviation programs and policies, such as micro-loans and microfinance [20, 21]. Pakistan has made stride in poverty reduction from 1991 to 2008 (before our studied period), reducing the poverty rate from 62% to 21% based on the international poverty line of 1.25 USD per day in 2005 PPP [22].

With the 2030 SDGs deadline on the horizon, many studies evaluate and compare different countries’ progress toward UHC [23–25]. Although following the same two domains stipulated in the SDGs 3.8.1 and 3.8.2, essential health services coverage and financial risk protection, previous studies diverge on the selection of specific indicators to measure the UHC progress [2, 3, 26], which creates a barrier to cross-country comparison. In efforts to harmonize the indicators and enable international comparison, Wagstaff et al. (2016) propose an index method to measure the UHC progress on a scale from 0 to 100, with 100 being the most progress toward UHC [24]. Although Wagstaff et al. (2020) covered Pakistan’s 2009 UHC index for international comparison, to our knowledge, no previous study has examined Pakistan’s UHC index over time and at a subnational level.

Recent development in the literature also highlights the need for subnational analysis, as regional inequality can hamper the progress toward UHC target in LMICs [26–28]. A subnational analysis can identify the provinces that are lagging behind in achieving UHC, which reflects the SDG principle of “Leave No One Behind”. Furthermore, in large countries with notable regional disparities, relying solely on national-level measures to gauge UHC progress might not provide a comprehensive understanding, because certain regions, for better or worse, can diverge significantly from the national average. We find several subnational UHC analyses in the literature, particularly in LMICs, such as Iraq, Vietnam, and Ghana [26–28]. However, to date, no such subnational analysis has been done for Pakistan. Subnational analysis is particularly relevant for Pakistan because of significant spatial health disparity, with a nine-year difference in life expectancy between Balochistan and Islamabad in 2019 [29]. Furthermore, Pakistan faces challenges with the maldistribution of medical professionals and significant understaffing in remote areas, which are notable weaknesses in the health care system [30].

Pakistan’s UHC program and its expansion can improve medical service coverage and financial risk protection at both national and subnational level. However, chronic government underinvestment in health care and persistent geographic socioeconomic inequality can be a major headwind for Pakistan’s UHC aspiration. In the background of these two competing forces, our retrospective study provides a comprehensive assessment of Pakistan’s progress toward UHC over time, at both national and subnational level.

## Methods

We build on the UHC index method proposed by Wagstaff et al. (2016) and Wagstaff & Neelsen (2020), while adapting the index components to the case of Pakistan. Similar to Wagstaff & Neelsen (2020), we assign equal weights to the two domains of UHC, medical service provision and financial risk protection. We further divide the medical service provision into two weighted subdomains: prevention (25%) and treatment (75%). The prevention subdomain consists of two equally weighted indicators: antenatal care coverage indicator is the percentage of women with at least four antenatal care visits; full immunization indicator is the percentage of children who have received one dose of BCG vaccine, three doses of DPT vaccine, three doses of polio vaccine, and one dose of Measles vaccine. We are not able to cover breast cancer screening and cervical cancer screening as Wagstaff & Neelsen (2020), due to the lack of data at subnational level. We include medical assistance at delivery, diarrhea treatment, acute respiratory infection (ARI) treatment, and inpatient admissions indicator in the treatment subdomain for the latest round of survey. Inpatient admissions indicator carries 50% weight, and the rest 50% weight would be equally shared by the other three indicators. For the early rounds of survey in which inpatient admissions data are not available, we adapt the UHC index formula and exclude inpatient admissions. The adapted treatment domain includes medical assistance at delivery, diarrhea treatment, and ARI treatment with equal weights. Medical assistance at delivery indicator is the percentage of women receiving medical assistance when giving birth. Diarrhea treatment indicator is the percentage of children with diarrhea symptoms receiving formal health care. ARI treatment indicator is the percentage of 15–23 months children with cough and rapid breathing symptoms receiving formal health care. Financial risk protection is measured by the share of population incurring CHE (10% or more of overall household consumption). All the indicators are calculated as the percentage of applicable population using the service, except for financial risk protection and inpatient admissions. Financial risk protection indicator is calculated as 100 minus the share of population incurring CHE. Inpatient admissions indicator is the percentage of population age 18 or older using inpatient care in the last 12 months and is normalized against the WHO benchmark of 10 admissions per 100 persons [31], which is equivalent to 9.03% of population reporting at least one inpatient admission in the past 12 months [24]. The definition for each indicator is elaborated in Appendix Table 1.

The UHC index is computed in the following three steps using the weights detailed above: first, we calculate the weighted geometric average for prevention service coverage subdomain and treatment service coverage subdomain using their respective indicators; then, we calculate the weighted geometric average for service coverage domain with the two subdomains above; finally, the UHC index is the weighted geometric average of the service coverage domain and financial risk protection domain with equal weights [24]. The UHC index is calculated at national level and subnational level for Pakistan for 2007, 2013, and 2018.

UHC index presents the population average at national or subnational level, which can mask the health inequality within the population. Moreover, a growing body of literature highlights how vulnerable populations may not fully benefit from the UHC progress as much as the index suggests [32, 33]. This is particularly relevant to Pakistan, where a significant portion of the population resides in rural areas. The maldistribution of medical resources and inadequate rural medical infrastructure can exacerbate health inequality. Therefore, we conduct inequality analysis using Concentration Index and Logistic regressions to complement the UHC index above, shedding light on the factors contributing to health inequality. First, we compute concentration index (CI) for each medical service coverage indicator to assess the level of inequality in utilization of medical services, including antenatal care, child immunization, medical assistance at delivery, ARI treatment, diarrhoea treatment, and inpatient admission (if available in the data). Then, we follow the World Bank guidelines and use probit regression for CI decomposition [34–36]. Wagstaff et al. (2003) show that concentration index can be decomposed with linear regression in which regressors are the concentration indices of the explanatory variables. Van Doorslaer et al. (2004) further develop the methodology and incorporate nonlinear regression (probit) into CI decomposition. The dependent variable in our CI decomposition analysis is a binary variable indicating the utilization of the corresponding medical services listed above (e.g., whether the child received full immunization). We include province, urban or rural household, mother’s age, wealth index, and education attainment (years of schooling) as independent variables for CI decomposition analysis. Wealth index is a composite score based on the ownership of various consumer goods in a household, which is readily available in the data. We also include health insurance coverage for 2018 analysis, as the variable is unavailable in earlier years.

For financial risk protection indicator, namely CHE, we use Logistic regression and Sartori’s two-step model to assess the main contributors to CHE [37]. CHE is a crucial metric for UHC, which is consistent with SDG indicator 3.8.2 financial risk protection [38]. All three years of data are pooled together for analysis. The dependent variable is a binary variable indicating whether household medical expenditure, when divided by total household expenditure, equals or exceeds 10%. Additionally, for robustness checks, we calculate an alternative version of CHE using a threshold of 25%. The independent variable of interest is wealth index in quintiles, while we control for age, sex, education, employment status of household head, share of household members under 5 and share of household members above 65. We also include a series of dummy variables to control for province and year fixed effects. The share of household members under 5 and above 65 can be a proxy for health care demand. We use Sartori’s two-step model to correct the potential bias arising from the two-stage selection of CHE (whether to seek health care and whether to incur CHE if seeking care). For example, some households, facing serious illness, may not seek medical service because of affordability issues. Therefore, their response in the dataset is zero medical expenditure and no CHE. However, they would have incurred CHE if they sought care. Their responses in the dataset should not be considered as true zero and warrants Sartori’s two-step model to correct the bias [37]. Sartori’s two-step model uses the same set of independent variables for both steps. The first step uses the full sample to run a regression with a binary variable (whether incurring medical expenditure) as dependent variable. The dependent variable for the second step regression is whether experiencing CHE, but the sample for the second step is those who incur medical expenditure. Both steps use Logistic regression.

## Data

We use datasets from the Demographic and Health Surveys (DHS) and Household Integrated Economic Survey (HIES). Both datasets can be downloaded for free from their respective websites [39, 40]. Data for medical service coverage indicators are from DHS which cover women between age 15 and 49 and children under 59 months. CHE indicator is from HIES which includes detailed and categorized expenditure and consumption data from the sampled households. Total household expenditure with follow-up correction is readily available in HIES and we calculate household medical expenditure by adding up expenditure in the relevant categories.

DHS datasets are available for 2006–2007, 2012–2013, and 2017–2018, so we match them with 2007–2008, 2013–2014, and 2018–2019 datasets from HIES, because HIES was not conducted in the same year as DHS. The datasets cover four provinces: Punjab, Khyber Pakhtunkhwa, Sindh, and Balochistan. Islamabad is included in Punjab province for data consistency between the two surveys. These provinces cover 97.6% of Pakistan’s total population according to the 2017 Population Census [41]. The other provinces, including Azad Jammu and Kashmir, Federally Administered Tribal Areas (FATA), and Gilgit Baltistan, are not covered by the survey, or the data is not released for security and political reasons [42]. We use publicly available secondary data as detailed above. Ethics approval is not needed for our study.

## Results

Table 1 reports UHC index and selected UHC indicators at national and subnational level over time. Panel A excludes the indicator for inpatient admissions due to the lack of data in earlier years, while Panel B includes it for a complete version of UHC index. Table 1 Panel A shows a steady progress toward UHC at national level, elevating from 67.6 in 2007, 74 in 2013, to 79.2 in 2018. The leading provinces (Punjab and Sindh) persistently have about a ten-point edge over the lagging provinces (Balochistan and KP) in Panel A. Panel B reports Pakistan’s UHC index at 71 in 2018. The difference between the leading and lagging provinces is slightly smaller in Panel B with the complete version of UHC index.

**Table 1:** UHC Index and selected UHC indicators at National and Subnational Level by Year

Year	Province	Antenatal care coverage	Full immunization	Medical assistance at delivery	Diarrhoea treatment	ARI treatment	Inpatient admissions	CHE at 10%	UHC index
Panel A									
2007	National level	28.8	44.8	39.2	63.9	72.6		90.5	67.6
2007	Punjab	29.7	50.7	38.2	63.8	73.7		89.7	67.9
2007	Sindh	36.8	35.9	45.0	69.3	75.3		96.1	72.0
2007	Khyber Pakhtunkhwa	18.2	40.4	38.1	58.2	65.6		80.5	59.9
2007	Balochistan	7.8	34.1	23.2	49.8	55.1		99.1	56.3
2013	National level	36.6	55.3	52.3	74.6	75.1		90.8	74.0
2013	Punjab	38.8	67.3	52.9	80.2	79.5		87.9	75.2
2013	Sindh	44.5	23.8	60.5	76.6	75.0		97.5	75.2
2013	Khyber Pakhtunkhwa	24.0	55.1	49.0	56.3	63.0		87.5	66.2
2013	Balochistan	12.3	24.8	18.0	58.6	52.5		98.0	55.4
2018	National level	51.7	68.8	66.1	69.6	79.4		91.7	79.2
2018	Punjab	56.9	86.8	69.5	74.3	83.6		91.0	82.2
2018	Sindh	54.2	44.6	71.6	72.1	80.5		96.0	80.4
2018	Khyber Pakhtunkhwa	45.0	56.8	59.9	58.4	71.2		86.0	71.6
2018	Balochistan	23.1	38.0	36.1	60.4	57.6		96.6	65.1
Panel B									
2018	National level	51.7	68.8	66.1	69.6	79.4	40.1	91.7	71.0
2018	Punjab	56.9	86.8	69.5	74.3	83.6	38.5	91.0	72.4
2018	Sindh	54.2	44.6	71.6	72.1	80.5	38.2	96.0	70.9
2018	Khyber Pakhtunkhwa	45.0	56.8	59.9	58.4	71.2	48.4	86.0	68.1
2018	Balochistan	23.1	38.0	36.1	60.4	57.6	42.7	96.6	63.2

**Fig. 1:**
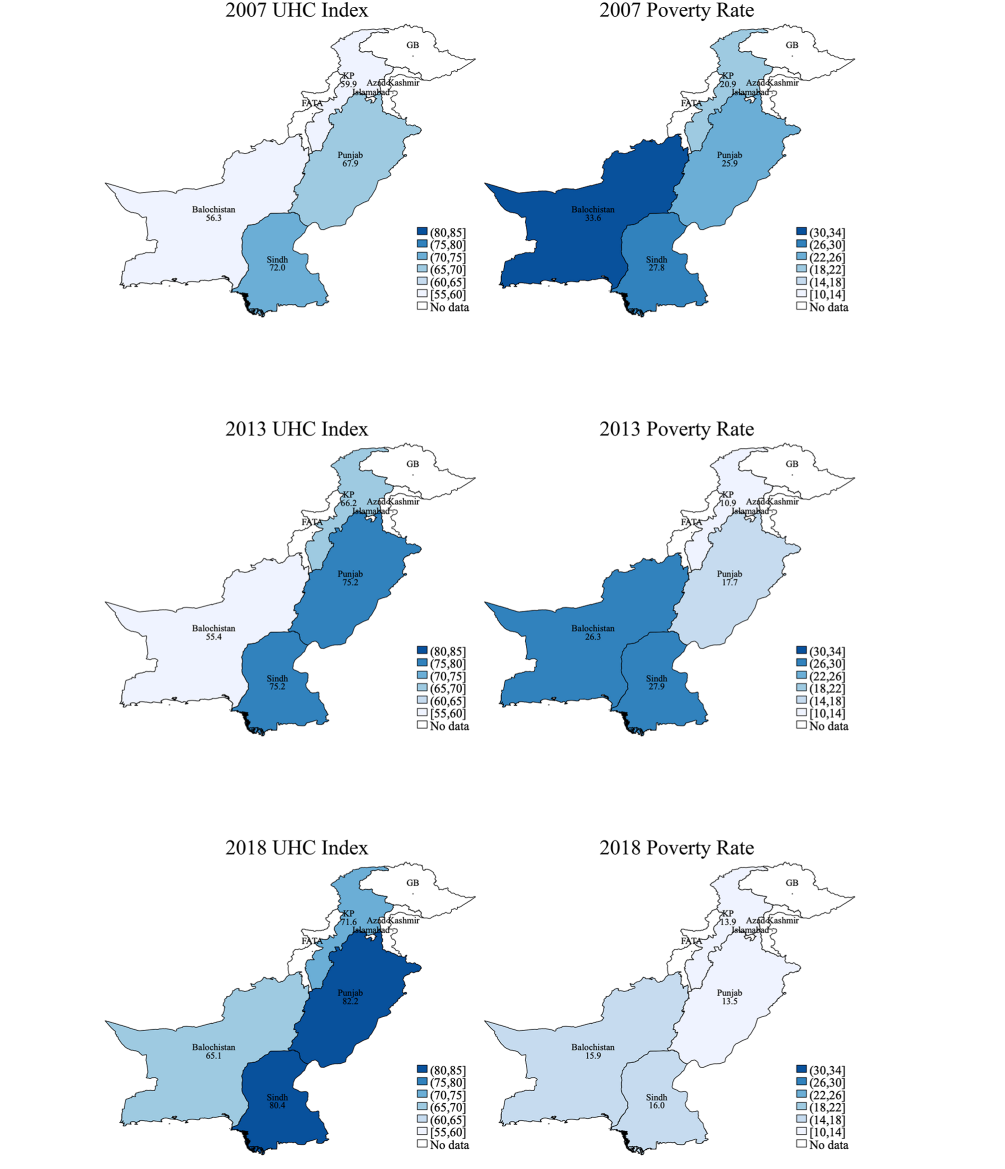
UHC index and poverty rate by province and year

We illustrate provincial UHC index on the maps in Fig. 1 and juxtapose them with additional maps of provincial poverty rate. We use poverty rate as a proxy for regional economic development to highlight the nexus between economic development and UHC progress. Darker blue indicates higher poverty rate in the poverty map and higher UHC index in the UHC map. The persistent difference in the shades of blue suggests deep-rooted between-province inequality in both UHC progress and poverty. Nevertheless, overall change in the color shows a collective progress in poverty reduction and UHC improvement.

**Fig. 2:**
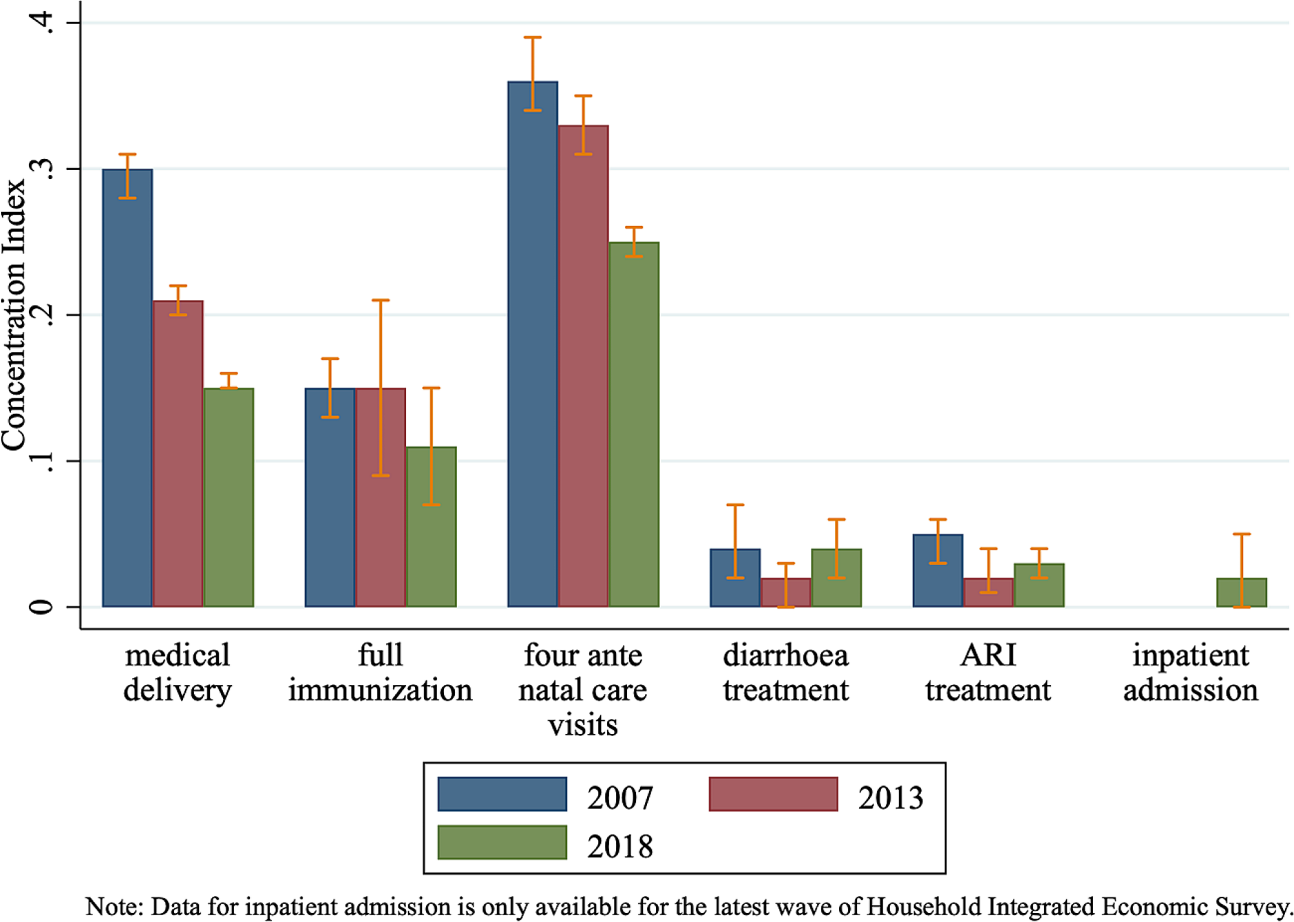
Concentration index for medical service coverage indicators by year

While UHC index measures the overall progress, Fig. 2 takes a different perspective and focuses on each specific component of the index, particularly through an inequality lens. We calculate the concentration index for each UHC indicator and illustrate them by year in Fig. 2, while the detailed CI analysis results are reported in Appendix Table 2. The positive yet shrinking bars suggest persistent yet declining pro-rich inequality in access to medical services. Meanwhile, the bars for medical assistance during delivery (CI = 0.30 in 2007) and having at least four antennal care visit (CI = 0.37 in 2007) are particularly and stubbornly high, suggesting continued serious inequality in access to maternal care.

The distribution of “zero-dose children” (children without any vaccination) corroborates the regional inequality in UHC progress but at an appalling level. Punjab reported less than 1% of the children without any vaccinations in 2018, while Balochistan reported 29% of the children never receiving any vaccinations (Appendix Table 3). At the national level, 4.7% of children have never received any vaccination. Moreover, the distribution of zero-dose children by wealth index (Appendix Fig. 1) suggests pro-rich inequality, which is similar to other UHC indicators we analyze above. This issue is particularly disturbing in Balochistan where more than half of the children in the lowest wealth index quintile have never received any vaccinations. In contrast, only 1% of the children in the lowest wealth index quintile in Punjab have never received any vaccinations (Appendix Fig. 2). We also observe a similar provincial disparity in the percentage of not-fully-vaccinated children in the lowest wealth quintile (Appendix Fig. 2). These findings support targeted vaccination campaigns to promote child immunization, particularly in Balochistan province and among the poor.

**Table 2:** Odds ratios from logistic regression on CHE (pooled)

	CHE at 10%	CHE at 25%
Wealth index quintile (1 as reference group)		
2	0.760***	0.847
	(0.0368)	(0.108)
3	0.620***	0.686***
	(0.0319)	(0.0897)
4	0.441***	0.475***
	(0.0268)	(0.0775)
5 (most wealthy)	0.313***	0.339***
	(0.0236)	(0.0680)
Household head sex, male	1.218***	1.585**
	(0.0832)	(0.285)
Household head age (80 or older as reference group)		
Younger than 30	1.246	1.488
	(0.198)	(0.564)
30–39	1.026	1.085
	(0.158)	(0.398)
40–49	0.989	1.150
	(0.151)	(0.415)
50–59	1.293*	1.509
	(0.197)	(0.542)
60–69	1.524***	1.650
	(0.223)	(0.545)
70–79	1.422**	1.362
	(0.214)	(0.453)
Household head employed	0.764***	0.577***
	(0.0415)	(0.0770)
Urban	1.104**	1.133
	(0.0450)	(0.112)
Number of household members	0.969***	0.934***
	(0.00653)	(0.0203)
Percent of household members older than 65	2.501***	2.869***
	(0.346)	(0.945)
Percent of household members younger than 5	1.672***	1.268
	(0.195)	(0.392)
Household head education (incomplete primary education as reference group)		
Primary	0.797***	1.033
	(0.0698)	(0.232)
Incomplete secondary	0.815**	0.828
	(0.0685)	(0.182)
Secondary	0.801**	0.717
	(0.0696)	(0.163)
Tertiary	0.808**	0.735
	(0.0770)	(0.188)
Other	0.816***	0.759
	(0.0600)	(0.146)
Province (Balochistan as reference group)		
KP	7.316***	4.632***
	(0.773)	(1.158)
Punjab	5.263***	4.103***
	(0.546)	(1.000)
Sindh	1.662***	0.953
	(0.183)	(0.263)
Year (2007–2008 as reference group)		
2018–2019	0.792***	0.524***
	(0.0374)	(0.0618)
2011–2012	0.916*	0.845
	(0.0451)	(0.0986)
N	57,769	57,769

Standard error is reported in parentheses. * *p* < 0.1, ** *p* < 0.05, *** *p* < 0.01

**Table 3:** Coefficients from Sartori’s two-step model (pooled)

	CHE at 10%		CHE at 25%	
	Selection	Outcome	Selection	Outcome
Wealth index quintile (1 as reference group)				
2	-0.0603	-0.140***	-0.0632	-0.0482
	(0.0868)	(0.0227)	(0.0868)	(0.0456)
3	-0.113	-0.251***	-0.113	-0.127***
	(0.0849)	(0.0241)	(0.0848)	(0.0483)
4	-0.296***	-0.433***	-0.296***	-0.276***
	(0.0810)	(0.0268)	(0.0809)	(0.0544)
5 (most wealthy)	-0.388***	-0.595***	-0.390***	-0.403***
	(0.0836)	(0.0319)	(0.0835)	(0.0645)
Household head sex, male	0.101	0.0951***	0.105	0.179***
	(0.0754)	(0.0309)	(0.0753)	(0.0613)
Household head age (80 or older as reference group)				
Younger than 30	0.299*	0.162**	0.303*	0.0885
	(0.170)	(0.0753)	(0.171)	(0.137)
30–39	0.239	0.0829	0.243	0.00483
	(0.160)	(0.0730)	(0.160)	(0.132)
40–49	0.198	0.0540	0.200	0.0106
	(0.157)	(0.0724)	(0.157)	(0.130)
50–59	0.154	0.190***	0.158	0.149
	(0.157)	(0.0724)	(0.157)	(0.130)
60–69	0.217	0.253***	0.216	0.177
	(0.151)	(0.0703)	(0.151)	(0.124)
70–79	0.149	0.188***	0.160	0.110
	(0.152)	(0.0719)	(0.152)	(0.126)
Household head employed	0.0442	-0.135***	0.0389	-0.217***
	(0.0620)	(0.0242)	(0.0618)	(0.0452)
Urban	-0.0453	0.0285	-0.0468	0.0354
	(0.0409)	(0.0179)	(0.0409)	(0.0357)
Number of household members	0.0462***	-0.0136***	0.0459***	-0.0222***
	(0.00868)	(0.00271)	(0.00866)	(0.00572)
Percent of household members older than 65	-0.518***	0.661***	-0.543***	0.578***
	(0.145)	(0.0687)	(0.146)	(0.116)
Percent of household members younger than 5	0.322**	0.303***	0.310**	0.134
	(0.152)	(0.0528)	(0.152)	(0.108)
Household head education (incomplete primary education as reference group)				
Primary	-0.0365	-0.133***	-0.0383	-0.0246
	(0.140)	(0.0403)	(0.140)	(0.0775)
Incomplete secondary	-0.210	-0.144***	-0.209	-0.0767
	(0.130)	(0.0390)	(0.130)	(0.0757)
Secondary	-0.208	-0.135***	-0.211	-0.0862
	(0.129)	(0.0397)	(0.129)	(0.0778)
Tertiary	-0.496***	-0.145***	-0.500***	-0.0674
	(0.127)	(0.0419)	(0.126)	(0.0827)
Other	-0.0703	-0.144***	-0.0710	-0.141**
	(0.124)	(0.0345)	(0.124)	(0.0671)
Province (Balochistan as reference group)				
KP	-0.107	0.977***	-0.104	0.584***
	(0.0934)	(0.0402)	(0.0934)	(0.0818)
Punjab	-0.201**	0.807***	-0.200**	0.529***
	(0.0852)	(0.0392)	(0.0852)	(0.0795)
Sindh	0.145	0.273***	0.148	0.0185
	(0.0957)	(0.0417)	(0.0957)	(0.0887)
Year (2007–2008 as reference group)				
2018–2019	0.459***	-0.0847***	0.463***	-0.164***
	(0.0479)	(0.0206)	(0.0479)	(0.0414)
2011–2012	0.407***	-0.0577***	0.408***	-0.0331
	(0.0518)	(0.0218)	(0.0518)	(0.0422)
N	57,772	57,772	57,772	57,772

Standard error is reported in parentheses. * *p* < 0.1, ** *p* < 0.05, *** *p* < 0.01

In efforts to assess the main contributors to health inequality, we conduct CI decomposition analysis for each medical service coverage indicator. The results are reported in Appendix Tables 4, 5, and 6 for 2007, 2013, and 2018, respectively. Household wealth index is the main contributor to inequality for all medical service coverage indicators. This is consistent with our findings above and again highlights the role of poverty in health care access. Province is another major contributor to inequality, particularly for inequality in treatment indicators (treatment for diarrhea and ARI). The prominence of province variable may be a reflection of supply side factors, such as availability and distribution of health care facilities and medical professionals. This finding also corroborates the between-province disparity in UHC progress. In addition to CHE, health insurance coverage is another commonly used measure for financial protection. We include health insurance coverage in the CI decomposition analysis for 2018, as it is unavailable in earlier years of datasets. The result in Appendix Table 6 suggests that health insurance coverage is not a significant contributor to inequality in health service utilization, which could be partly attributed to the low insurance coverage that only 2% of the surveyed individuals are insured.

Table 2 reports odds ratios and confidence intervals from a pooled repeated cross-sectional analysis while accounting for sample weight. The complete-case analysis has a sample size of 57,769. The dependent variable is CHE at 10% threshold, as shown in Column (1) The main explanatory variable is wealth index expressed in quintile. We control for a series of household characteristics, province dummies, and year dummies. Household characteristics include sex, age, education attainment, employment status of household head, urban or rural location, as well as the percentage of household members older than 65 and younger than 5. As expected, households with higher percentage of household members older than 65 and younger than 5 are more likely to incur CHE, because the elderly and the very young have higher need for care. Furthermore, households with formally employed household head are less likely to incur CHE. We also perform a sensitivity analysis using an alternative CHE threshold of 25%. The results are similar, as reported in Column (2) Moreover, we also conduct the same analysis separately for each year and the results are reported in Appendix Table 7.

We advance the analysis with Sartori’s two-step model to account for potential bias that households with limited financial resources may not seek medical service at all. Table 3 shows log odds ratios and confidence intervals from a pooled repeated cross-sectional analysis with Sartori’s two-step model, while accounting for sample weight. The complete-case analysis has a sample size of 57,772. Column 1 reports results from the selection stage (whether to seek care or not) and Column 2 reports results for CHE conditional on incurring medical expenditure. Sartori’s two-step model has a few additional findings, but the conclusion is similar with that from Logistic regression. Formal employment of the household head is not associated with whether to seek health care or not, but negatively associated with incurring CHE conditional on seeking health care. This finding is consistent with the literature and might be explained by the provision of workplace health insurance that provides financial risk protection against CHE [43, 44]. Furthermore, households with high wealth index (top two quintiles) are less likely to seek medical care, which may suggest a better health status and lower demand for medical services. Similar to the results from the Logistic regression, households in top wealth index quintiles are less likely to incur CHE. Moreover, the percentage of household members over 65 is negatively correlated with seeking health care, but positively correlated with incurring CHE conditional on seeking care. We also conduct the same analysis separately for each year and the results are reported in Appendix Table 8. Further descriptive analysis shows that medicine expenditure is the top contributor to health care spending and the share of medicine in health care expenditure has been increasing. For households with CHE, about 70% of the health care spending was on medicines in 2018, rising from 41% in 2013 and 48% in 2007.

## Discussion

Our national level analysis highlights Pakistan’s steady progress toward UHC, yet the subnational analysis suggests deep-rooted inequality in UHC progress among provinces. Pakistan’s UHC index stands at 71 (Table 1 Panel B), which highlights Pakistan’s efforts in UHC-inspired reforms and nationwide expansion of UHC programs in recent years. The increase in UHC index is largely attributed to improvement in medical assistance at delivery and antenatal care coverage. Pakistan increased the budget for Lady Health Workers (LHWs) Programme between 2003 and 2008, which supported the expansion of LHWs staff size from 70,000 to 100,000 [17]. The increased funding for and the expansion of LHWs are particularly beneficial for child and maternal care, as reflected in the increase in medical assistance at delivery and antenatal care coverage. Furthermore, the substantial increase in UHC index from 2013 to 2018 also coincides with Pakistan’s rollout and subsequent expansion of UHC programs since 2015. Although a few retrospective studies have examined the progress toward UHC in other LMICs, their construction of UHC index and studied period are different from ours, which does not allow a fair peer country comparison of UHC progress in the same time frame.

Behind the nationwide progress toward UHC is the persistent inequality among provinces. To put it into perspective, if the provinces were independent countries, the UHC index of the leading province, Punjab, would be similar to upper-middle-income countries, such as Indonesia, although Punjab’s per capita GDP would still fall under lower-middle income economies by the World Bank classification [6, 45]. Meanwhile, the UHC index of the lagging province, Balochistan, would be similar to lower-middle-income countries, such as Bangladesh. Although all provinces have made significant advancements in UHC, we also observe a provincial disparity that the leading province, Punjab, increases 14 points in UHC index from 2007 to 2018, while the lagging province, Balochistan, only increases 9 points.

Pakistan’s progress toward UHC is in tandem with the economic development and poverty reduction, as shown in the maps in Fig. 1. The poverty maps in Fig. 1 suggest stubborn provincial disparity in economic development, which eclipses the fast nationwide reduction in poverty. Provinces with lower poverty rate, such as Punjab, constantly have higher UHC index. This finding highlights provincial economic underdevelopment as a key barrier on Pakistan’s path toward UHC. We also underscore the the synergy of poverty alleviation and UHC program, as poverty alleviation efforts are particularly needed in the least developed provinces.

At household level, our analysis also shows a positive correlation between poverty and incurring CHE, as demonstrated by the negative association between incurring CHE and household wealth index. Our finding is confirmed by existing studies from other low-income countries. The household living standard survey in Nigeria shows that poorer households are more likely to incur CHE [46]. A previous study in Myanmar also suggests that households in the lowest socioeconomic quintile are more likely to incur CHE [47]. Similarly, another study in Burkina Faso shows households in higher income quintile are less likely to incur CHE [48]. Our study can further contribute to this emerging literature in the context of low- and middle-income countries.

Our CI analysis highlights the pro-rich inequality in access to medical services, which is common in LMICs and supported by many studies. A systematic review on maternal care inequality in LMICs shows more affluent women are more likely to use maternal care [49]. A series of country-specific studies also confirm the positive association between maternal care utilization and socioeconomic status, including the neighboring country of Bangladesh [50–52]. On the other hand, our analysis also shows a decreasing CI for virtually all indicators of medical service coverage, which suggests that the pro-rich inequality is declining.

Furthermore, the CI decomposition results also corroborate our findings from UHC index. All three years of CI decomposition analyses show that household socioeconomic status is the key contributor to inequality in essential health service utilization, which mirrors our subnational level UHC index analysis that provincial economic development is correlated with provincial UHC index. Provinces with lower poverty rate report higher UHC index. Surprisingly, health insurance coverage is not a significant contributor to inequality in essential health service utilization for 2018. Further descriptive analysis shows that less than 3% of the individuals in the sample have health insurance. Pakistan was still rolling out and expanding UHC program at the time of survey, so the findings could be different if newer survey data become available.

We would like to emphasize shortcomings in Pakistan’s health care landscape that hinder progress toward UHC. One notable issue is the significant portion of health care expenditure allocated to medicines, which surged to 70% in 2018. More disturbingly, the share of medicine expenditure as a percentage of overall health spending increased 39 percentage points from 2007 to 2018, which suggests the current UHC program and its expansion have not addressed medicine expenditure issue. These staggering numbers underscore the necessity for broader medicine coverage within the UHC program and additional policy measures to contain medicine costs.

Our findings on medicine expenditure align with the existing literature, which shows that out-of-pocket spending on medicines constitutes a substantial, if not the predominant, portion of household health expenditure in LMICs. A household survey in Ethiopia shows that out-of-pocket medicine expenditure remains the largest contributor to health care spending, staying above 65% for all years of survey from 2010 to 2016 [53]. A study in Tajikistan shows that more than three fourths of health care spending is on medicines [54].

Child immunization remains a key weak point among the UHC indicators. In the latest DHS data (2018), about one third of the children are not fully vaccinated against BCG, DPT, polio, and measles. More disturbingly, about one sixth of these not-fully-vaccinated children have never received any vaccinations at all (zero-dose children), which underscores the challenge that Pakistan faces in promoting child immunization. The high percentage of children with incomplete or zero vaccination also allows vaccine-preventable diseases, such as polio, to persist as endemic in Pakistan. After more than two decades of declining polio cases since the introduction of Expanded Program on Immunization (EPI) in 1978, Pakistan’s number of polio cases started to plateau in early 2000s [55]. Pakistan reported 146 polio cases in 2019, joining Afghanistan as the only two countries in the world with polio endemic [56]. These avoidable public health tragedies highlight the urgent need for targeted vaccination campaigns to promote routine immunization.

Our study is subject to certain limitations related to data availability and methodology. Firstly, the most recent data utilized in our analysis is from 2018, which falls within the period of Pakistan’s ongoing expansion of UHC initiatives. For instance, it predates the rollout of the UHC program in Punjab province in late 2020 [18]. Consequently, our estimates of the UHC index using this data may potentially underestimate the impact of the UHC program. Additionally, the COVID-19 pandemic may have disrupted medical service delivery, including child immunization, which could have adverse effects on Pakistan’s progress toward achieving UHC. We are unable to conduct a comprehensive evaluation due to the absence of recent data. Moreover, both datasets utilized in our analysis are based on repeated cross-sectional data, which means we cannot follow the medical expenditure of the same households over time. Additionally, inpatient admissions data is unavailable for earlier survey years, precluding its inclusion for cross-year comparisons. Lastly, our survey datasets span over a decade, and survey questions have evolved over time. Consequently, some variables may have comparability issues, despite our efforts to harmonize them to the best of our ability.

In terms of methodology, our regression analyses only suggest correlations and cannot establish causal relationships. Additionally, the concentration index approach has inherent limitations, such as restricted bounds of CI when the variable of interest is binary [57]. While this could pose challenges for cross-country comparisons, it is less of a concern for our study, which focuses solely on Pakistan. Furthermore, our analysis reveals a consistent improvement in medical service coverage indicators alongside an overall reduction in CI, which helps mitigate concerns regarding CI bounds.

## Conclusion

We employ the established UHC index methodology to assess Pakistan’s advancement toward UHC on both national and subnational scales. Despite the mounting challenges of economic underdevelopment and endemic disease burdens, Pakistan’s policy initiatives in primary care provision, along with the provincial governments’ efforts in piloting and expanding UHC programs, have yielded a consistent increase in the UHC index. However, provincial economic underdevelopment, as indicated by provincial poverty levels, remains a significant obstacle to Pakistan’s UHC progress. Provinces with higher poverty rates consistently lag behind in nearly all UHC indicators, resulting in a nearly ten-point disparity in the UHC index across all three years analyzed. Our findings underscore several policy recommendations aimed at expediting Pakistan’s pathway toward UHC. Firstly, government authorities should increase the income threshold of the existing UHC program to cover a broader segment of vulnerable population, rather than solely targeting the population below the poverty line. Secondly, sustained investment and policy continuity are essential for health care reforms. Government underfunding remains a significant weakness in Pakistan’s health care system and must be addressed to ensure the effectiveness and sustainability of UHC initiatives. Lastly, it’s imperative for government authorities to align the UHC program with broader goals of economic development and poverty alleviation, as these efforts are mutually reinforcing. By integrating UHC expansion with poverty reduction strategies, Pakistan can foster comprehensive improvements in health care access and overall societal well-being.

It may also be pertinent to recommend to organizations such as the United Nations (UN) to consider measuring progress toward UHC not only at the national level but also at the regional level. By examining UHC progress on a regional basis, policymakers can gain insights into disparities within countries and tailor interventions to address specific challenges faced by different regions. This approach could lead to more targeted and effective strategies for advancing UHC and improving health care access for all populations, especially for countries with bottom-up policy formulation where local government are taking initiative for UHC programs and policies.

### Electronic supplementary material

Below is the link to the electronic supplementary material.

Supplementary Material 1

Supplementary Material 2

## Data Availability

The datasets are available on the DHS program website and Pakistan Bureau of Statistics website (https://www.dhsprogram.com/data/available-datasets.cfm?ctryid=31, https://www.pbs.gov.pk/content/microdata).

## Abbreviations

CHE: Catastrophic health expenditure
CI: Concentration index
LHWs: Lady Health Workers
PPP: Purchasing power parity
UHC: Universal health coverage
