# Supplementary material for: Pakistan’s path to universal health coverage: national and regional insights
Source: Int J Equity Health. 2024 Aug 15;23:162. doi: 10.1186/s12939-024-02232-1 (PMC11325752; doi:10.1186/s12939-024-02232-1)
Supplement: Supplementary file 1 — Supplementary Material 1 [file 12939_2024_2232_MOESM1_ESM.docx]

**Supplementary Material**

[Data Cleaning and Variable Construction 2](#_Toc169876830)

[Technical Details for Statistical Analysis 3](#_Toc169876831)

[UHC Index Construction 3](#_Toc169876832)

[Concentration Index and its Decomposition 4](#_Toc169876833)

[Logistic Regression and Sartori’s Two-Step Model 5](#_Toc169876834)

[Reference 6](#_Toc169876835)

Data Cleaning and Variable Construction

Household Integrated Economic Survey (HEIS) is matched to the nearest year of Demographic and Health Surveys (DHS) to construct the analytic sample for 2007, 2013, and 2018. Among the UHC index component indicators, antenatal care coverage, full immunization for children, medical assistance at delivery, diarrhea treatment, and acute respiratory infection treatment are from DHS. Inpatient admissions and catastrophic health expenditure are from HEIS. All indicators are expressed in percentage of application population using the medical service or incurring CHE. CHE is defined as total health expenditure taking up 10% or more of aggregated household consumption by monetary value. Aggregated household consumption is calculated by Pakistan Bureau of Statistics and available in the data. We compile total health expenditure by adding up all items of medical spending in the data and harmonize between different rounds of HEIS survey, as the included items and their definitions can change over time. For example, the 2007 HEIS questionnaire has a medical spending item for “purchase of medicines & vitamins, medical apparatus, and other equipment / supplies etc.” This item is broken down into multiple separate items in 2018 questionnaires: “Medicines/ Tablets/ Syrups etc.” and “Neck Braces/ Cotton Bandage/ Thermometer/ Blood Pressure Machine/ Other Medicine/Apparatus/ Eye Glasses.” These differences are not a problem for our analysis because we aggregate them into total health expenditure.

For our CI decomposition analysis, we use DHS data, and all independent variables are readily available in the data except province variable. Province variable is harmonized between DHS and HEIS that Islamabad is included in Punjab province. We use HEIS data for Logistic regression and Sartori’s two-step model. The dependent variable is incurring CHE, and the CHE variable construction is the same as above. The independent variable is household wealth index which we construct from a series of household assets ownership variables (such as livestock, TV, and fridge), housing characteristics variables (such as water supply and sewage management), and household finance variables (such as having a bank account and owning gold). We further convert the wealth index into quintiles 1-5, with 5 being the poorest. All regression covariates are readily available in the data except household head’s age and education attainment, share of household members under 5, and share of household members above 65. Age is converted into under 30, 30-39, 40-49, 50-59, 60-69, 70-79, 80 and above age groups. The 80 and above age group is used as reference group. Education attainment is classified into (i) incomplete primary education or no schooling, (ii) completed primary education, (iii) incomplete secondary education, (iv) completed secondary education, (v) all levels above secondary education, completed or not, (vi) other, including missing values. Group (i) incomplete primary education or no schooling is used as reference group. Shares of household members under 5 and above 65 capture the health care need from medically vulnerable household members. Age 5 and 65 are included during calculation.

Technical Details for Statistical Analysis

UHC Index Construction

UHC index formula uses geometric average and follows the form of a Cobb-Douglas function to allow tradeoff between different indicators at a diminishing rate [1], as policymakers can prioritize certain medical service over others, or prioritize service coverage over financial protection, vice versa. The functional form is the following:

$$UHC\equiv{Service Coverage}^{0.5}{Financial Protection}^{0.5}$$

Financial protection is exhibited as 100 minus the percentage of individuals incurring catastrophic health expenditure (CHE), as explained in the paper. Therefore, lower percentage of individuals incurring CHE would translate into better financial protection, which in turn increases UHC index.

We follow Wagstaff & Neelsen (2020) to select the indicators for UHC index construction. The selected indicators are corroborated by the existing literature and another similar UHC index constructed by the World Health Organization [2,3]. The main constraint for indicator selection is data availability. For example, breast and cervical cancer screening indicators are not available in the data. Furthermore, inpatient admissions data are only available in the latest round of survey. Therefore, we construct a complete version of UHC index with inpatient admissions for 2018 to provide the latest reading of UHC index, while constructing a revised version of UHC index without inpatient admissions for all three years to reveal the trend in UHC index.

# Concentration Index and its Decomposition

Concentration Index (CI) is commonly used to measure the degree of socioeconomic inequality. Methodologically, CI is derived from concentration curve and the absolute value of CI equals to twice the area between concentration curve and a 45-degree line. Therefore, CI can range from -1 to 1. A positive CI suggests pro-rich inequality while a negative CI indicates pro-poor inequality. A larger absolute value of CI means higher degree of inequality. We use the “conindex” command in Stata to calculate CI for each medical service coverage indicator. CI and its confidence interval are automatically calculated by the Stata command, and we plot them in Figure 2. CI decomposition shows the inequality contribution of a series of predictors, including province, urban or rural location, mother’s age, mother’s education, wealth index in quintile, and health insurance coverage if available in data. Each indicator’s contribution to CI is standardized to the percentage format. We follow the detailed Stata code from the World Bank to calculate and standardize contribution to CI [4]. Both CI calculation and CI decomposition account for sample weight.

# Logistic Regression and Sartori’s Two-Step Model

We run a Logistic regression using “logistic” command in Stata with the robust standard error option. Odds ratios and robust standard errors are reported in Table 3. Sartori’s two-step model is suitable for binary dependent variables that involves two-stage choices [5]. In the example of catastrophic health expenditure, an individual would first choose whether to seek health care or not. If he/she chooses to get care, he/she needs to make a second choice on intensity of care (whether to incur CHE or not). For individuals not seeking care because of affordability issue, they would not incur CHE, but this is not a true zero and would bias the estimate. Therefore, Sartori’s two-step model is a better choice for CHE modeling. Both steps of Sartori model use Logistic regression. The first step models the choice of seeking care or not, so the dependent variable is a binary variable indicating whether medical expenditure is above zero (seeking care). The second step models the intensity of care if seeking care, so the dependent variable is a binary variable indicating whether incurring CHE and the regression sample excludes those who have zero medical expenditure. Compared to the Heckman selection model, Sartori’s two-step model use the same set of explanatory variables for both steps.

Both Logistic regression and Sartori’s two-step model analysis use the same dependent and independent variables. CHE is defined as more than 10% (inclusive) of annual household consumption. We also use an alternative threshold of 25% for sensitivity analysis. We pool together three years of data for both Logistic regression and Sartori’s two-step model analysis, while including a series of dummies to control for the year of survey. All regressions account for sample weight. We use Sartori’s Stata package to run the model analysis. The package can be downloaded from the author’s website (https://asartori.mit.edu/research).

Reference

1. Wagstaff A, Neelsen S. A comprehensive assessment of universal health coverage in 111 countries: a retrospective observational study. Lancet Glob Health. 2020;8:e39–49.

2. World Health Organization. UHC Service Coverage Index (SDG 3.8.1) [Internet]. 2023 [cited 2023 Aug 25]. Available from: https://www.who.int/data/gho/data/themes/topics/service-coverage

3. Hogan DR, Stevens GA, Hosseinpoor AR, Boerma T. Monitoring universal health coverage within the Sustainable Development Goals: development and baseline data for an index of essential health services. The Lancet Global Health. 2018;6:e152–68.

4. O’Donnell O, Van Doorslaer E, Wagstaff A, Lindelow M. Analyzing health equity using household survey data: A guide to techniques and their implementation. The World Bank; 2008.

5. Sartori AE. An Estimator for Some Binary-Outcome Selection Models Without Exclusion Restrictions. Political Analysis. 2017/01/04. 2003;11:111–38.
