# Supplementary material for: Pakistan’s path to universal health coverage: national and regional insights
Source: Int J Equity Health. 2024 Aug 15;23:162. doi: 10.1186/s12939-024-02232-1 (PMC11325752; doi:10.1186/s12939-024-02232-1)

# Appendix Table 1. Component Indicators of UHC Index

| UHC index | Domain's share in the UHC index | Domains | Subdomain's share in the UHC dimensions | Subdomain | Indicator's share in the respective subdomain | Revised Indicator's share in the respective subdomain | Indicator | Indicator definition |
| --- | --- | --- | --- | --- | --- | --- | --- | --- |
|  | 50% | Medical service coverage | 25% | Prevention | 50% | 50% | At least 4 antenatal care visits | Percentage of women having at least four antenatal care visits |
|  |  |  |  |  | 50% | 50% | Full vaccination for children | Percentage of children who have received one dose of BCG vaccine, three doses of DPT vaccine, three doses of polio vaccine, and one dose of Measles vaccine |
|  |  |  | 75% | Treatment | 33.3% | 16.7% | Medical assistance at delivery | Percentage of women receiving medical assistance when giving birth |
|  |  |  |  |  | 33.3% | 16.6% | Treatment for Acute Respiratory Infection (ARI) | Percentage of 15-23 months children with cough and rapid breathing symptoms receiving formal health care |
|  |  |  |  |  | 33.3% | 16.6% | Treatment for diarrhea | Percentage of children with diarrhea symptoms receiving formal health care |
|  |  |  |  |  | 0% | 50% | Inpatient admission in last 12 months (% of population age 18 and older) | Percentage of population age 18 and older using inpatient care in the last 12 months. |
|  | 50% | Financial risk protection | 100% | Catastrophic health expenditure | 100% | 100% | Catastrophic medical expenditure at 10% or higher | Percentage of households with out-of-pocket health expenditure exceeding 10% of household consumption. |
| Note: the selection and definition of indicators in the table are based on Nikoloski et al. (2021) | | | | | | | | |

#

# Appendix Table 2. Concentration Index for Medical Service Coverage Indicators by Year

| Year |  | Coefficient | Robust standard error | t | p>t | 95% confidence interval | |
| --- | --- | --- | --- | --- | --- | --- | --- |
| 2007 | Medical delivery | 0.30 | 0.01 | 40.69 | 0.00 | 0.28 | 0.31 |
| 2007 | Full immunization | 0.15 | 0.01 | 19.06 | 0.00 | 0.13 | 0.17 |
| 2007 | Four ante natal care visits | 0.36 | 0.01 | 27.99 | 0.00 | 0.34 | 0.39 |
| 2007 | Diarrhoea treatment | 0.04 | 0.01 | 3.80 | 0.00 | 0.02 | 0.07 |
| 2007 | ARI treatment | 0.05 | 0.01 | 6.35 | 0.00 | 0.03 | 0.06 |
| 2007 | Inpatient admission |  |  |  |  |  |  |
| 2013 | Medical delivery | 0.21 | 0.01 | 35.36 | 0.00 | 0.20 | 0.22 |
| 2013 | Full immunization | 0.15 | 0.03 | 5.19 | 0.00 | 0.09 | 0.21 |
| 2013 | Four ante natal care visits | 0.33 | 0.01 | 33.10 | 0.00 | 0.31 | 0.35 |
| 2013 | Diarrhoea treatment | 0.02 | 0.01 | 2.14 | 0.03 | 0.00 | 0.03 |
| 2013 | ARI treatment | 0.02 | 0.01 | 3.98 | 0.00 | 0.01 | 0.04 |
| 2013 | Inpatient admission |  |  |  |  |  |  |
| 2018 | Medical delivery | 0.15 | 0.00 | 34.26 | 0.00 | 0.15 | 0.16 |
| 2018 | Full immunization | 0.11 | 0.02 | 5.37 | 0.00 | 0.07 | 0.15 |
| 2018 | Four ante natal care visits | 0.25 | 0.01 | 35.10 | 0.00 | 0.24 | 0.26 |
| 2018 | Diarrhoea treatment | 0.04 | 0.01 | 3.29 | 0.00 | 0.02 | 0.06 |
| 2018 | ARI treatment | 0.03 | 0.01 | 4.75 | 0.00 | 0.02 | 0.04 |
| 2018 | Inpatient admission | 0.02 | 0.01 | 1.73 | 0.08 | 0.00 | 0.05 |

| Appendix Table 3. Percentage of Children without Any Vaccination by Province and Year | | | |
| --- | --- | --- | --- |
| Province | 2007 | 2013 | 2018 |
| National level | 5.2% | 10.9% | 4.7% |
| Punjab | 2.0% | 9.7% | 0.7% |
| Sindh | 5.4% | 8.9% | 6.8% |
| Khyber Pakhtunkhwa | 12.0% | 12.6% | 7.2% |
| Balochistan | 25.7% | 31.8% | 28.7% |

# Appendix Table 4. Concentration Index Decomposition (2007)

|  |  | Elasticity | Concentration Index | Contribution | Percentage contribution | p-value |
| --- | --- | --- | --- | --- | --- | --- |
| Medical assistance during delivery | Province | 0.16 | -0.04 | -0.01 | -0.02 | 0.00 |
|  | Urban | 0.07 | 0.44 | 0.03 | 0.10 | 0.00 |
|  | Mother's age | -0.35 | 0.01 | 0.00 | -0.01 | 0.00 |
|  | Wealth index | 0.79 | 0.27 | 0.21 | 0.72 | 0.00 |
|  | Mother's education | 0.13 | 0.54 | 0.07 | 0.24 | 0.00 |
|  |  |  |  |  |  |  |
|  | Residual |  |  |  | -0.03 |  |
| Full immunization | Province | -0.15 | -0.04 | 0.01 | 0.04 | 0.00 |
|  | Urban | -0.01 | 0.44 | 0.00 | -0.02 | 0.53 |
|  | Mother's age | 0.60 | 0.01 | 0.00 | 0.02 | 0.00 |
|  | Wealth index | 0.42 | 0.27 | 0.11 | 0.76 | 0.00 |
|  | Mother's education | 0.05 | 0.54 | 0.03 | 0.20 | 0.00 |
|  |  |  |  |  |  |  |
|  | Residual |  |  |  | 0.00 |  |
| Four antenatal care visits | Province | -0.08 | -0.04 | 0.00 | 0.01 | 0.06 |
|  | Urban | 0.09 | 0.44 | 0.04 | 0.11 | 0.00 |
|  | Mother's age | -0.06 | 0.01 | 0.00 | 0.00 | 0.67 |
|  | Wealth index | 0.83 | 0.27 | 0.22 | 0.62 | 0.00 |
|  | Mother's education | 0.14 | 0.54 | 0.08 | 0.22 | 0.00 |
|  |  |  |  |  |  |  |
|  | Residual |  |  |  | 0.05 |  |
| Treatment for diarrhoea | Province | -0.04 | -0.04 | 0.00 | 0.04 | 0.25 |
|  | Urban | 0.01 | 0.44 | 0.01 | 0.15 | 0.37 |
|  | Mother's age | -0.02 | 0.01 | 0.00 | 0.00 | 0.87 |
|  | Wealth index | 0.10 | 0.27 | 0.03 | 0.64 | 0.07 |
|  | Mother's education | 0.01 | 0.54 | 0.01 | 0.14 | 0.38 |
|  |  |  |  |  |  |  |
|  | Residual |  |  |  | 0.03 |  |
| Treatment for ARI | Province | -0.06 | -0.04 | 0.00 | 0.05 | 0.01 |
|  | Urban | -0.02 | 0.44 | -0.01 | -0.17 | 0.08 |
|  | Mother's age | -0.16 | 0.01 | 0.00 | -0.02 | 0.02 |
|  | Wealth index | 0.23 | 0.27 | 0.06 | 1.30 | 0.00 |
|  | Mother's education | -0.02 | 0.54 | -0.01 | -0.17 | 0.05 |
|  |  |  |  |  |  |  |
|  | Residual |  |  |  | 0.01 |  |

# Appendix Table 5. Concentration Index Decomposition (2013)

|  |  | Elasticity | Concentration Index | Contribution | Percentage contribution | p-value |
| --- | --- | --- | --- | --- | --- | --- |
| Medical assistance during delivery | Province | -0.01 | -0.06 | 0.00 | 0.00 | 0.71 |
|  | Urban | 0.01 | 0.50 | 0.01 | 0.03 | 0.28 |
|  | Mother's age | -0.31 | 0.01 | 0.00 | -0.01 | 0.00 |
|  | Wealth index | 0.53 | 0.27 | 0.14 | 0.69 | 0.00 |
|  | Mother's education | 0.13 | 0.51 | 0.07 | 0.33 | 0.00 |
|  |  |  |  |  |  |  |
|  | Residual |  |  |  | -0.03 |  |
| Full immunization | Province | -0.26 | -0.06 | 0.02 | 0.11 | 0.00 |
|  | Urban | 0.02 | 0.50 | 0.01 | 0.07 | 0.66 |
|  | Mother's age | -0.66 | 0.01 | -0.01 | -0.04 | 0.09 |
|  | Wealth index | 0.21 | 0.27 | 0.06 | 0.37 | 0.20 |
|  | Mother's education | 0.13 | 0.51 | 0.07 | 0.45 | 0.00 |
|  |  |  |  |  |  |  |
|  | Residual |  |  |  | 0.04 |  |
| Four antenatal care visits | Province | -0.08 | -0.06 | 0.01 | 0.02 | 0.02 |
|  | Urban | 0.10 | 0.50 | 0.05 | 0.15 | 0.00 |
|  | Mother's age | -0.33 | 0.01 | 0.00 | -0.01 | 0.01 |
|  | Wealth index | 0.67 | 0.27 | 0.18 | 0.55 | 0.00 |
|  | Mother's education | 0.19 | 0.51 | 0.10 | 0.29 | 0.00 |
|  |  |  |  |  |  |  |
|  | Residual |  |  |  | 0.01 |  |
| Treatment for diarrhea | Province | -0.19 | -0.06 | 0.01 | 0.68 | 0.00 |
|  | Urban | 0.00 | 0.50 | 0.00 | 0.06 | 0.87 |
|  | Mother's age | -0.15 | 0.01 | 0.00 | -0.08 | 0.12 |
|  | Wealth index | 0.04 | 0.27 | 0.01 | 0.58 | 0.43 |
|  | Mother's education | -0.01 | 0.51 | 0.00 | -0.18 | 0.36 |
|  |  |  |  |  |  |  |
|  | Residual |  |  |  | -0.06 |  |
| Treatment for ARI | Province | -0.14 | -0.06 | 0.01 | 0.39 | 0.00 |
|  | Urban | 0.00 | 0.50 | 0.00 | -0.02 | 0.90 |
|  | Mother's age | -0.04 | 0.01 | 0.00 | -0.02 | 0.53 |
|  | Wealth index | 0.07 | 0.27 | 0.02 | 0.85 | 0.03 |
|  | Mother's education | -0.01 | 0.51 | 0.00 | -0.18 | 0.26 |
|  |  |  |  |  |  |  |
|  | Residual |  |  |  | -0.02 |  |

| Appendix Table 6. Concentration Index Decomposition (2018) | | | | | | |
| --- | --- | --- | --- | --- | --- | --- |
|  |  | Elasticity | Concentration Index | Contribution | Percentage contribution | p-value |
| Medical assistance during delivery | Province | -0.03 | -0.09 | 0.00 | 0.02 | 0.00 |
|  | Urban | 0.02 | 0.43 | 0.01 | 0.07 | 0.00 |
|  | Mother's age | -0.23 | 0.00 | 0.00 | -0.01 | 0.00 |
|  | Wealth index | 0.37 | 0.27 | 0.10 | 0.65 | 0.00 |
|  | Mother's education | 0.10 | 0.47 | 0.05 | 0.30 | 0.00 |
|  | Health insurance | 0.00 | -0.01 | 0.00 | 0.00 | 0.75 |
|  | Residual |  |  |  | -0.04 |  |
| Full immunization | Province | -0.26 | -0.09 | 0.02 | 0.21 | 0.00 |
|  | Urban | -0.08 | 0.43 | -0.03 | -0.29 | 0.01 |
|  | Mother's age | -0.09 | 0.00 | 0.00 | 0.00 | 0.69 |
|  | Wealth index | 0.35 | 0.27 | 0.10 | 0.84 | 0.00 |
|  | Mother's education | 0.05 | 0.47 | 0.02 | 0.20 | 0.12 |
|  | Health insurance | 0.00 | -0.01 | 0.00 | 0.00 | 0.97 |
|  | Residual |  |  |  | 0.04 |  |
| Four antenatal care visits | Province | -0.05 | -0.09 | 0.00 | 0.02 | 0.02 |
|  | Urban | 0.04 | 0.43 | 0.02 | 0.07 | 0.00 |
|  | Mother's age | -0.10 | 0.00 | 0.00 | 0.00 | 0.28 |
|  | Wealth index | 0.62 | 0.27 | 0.17 | 0.68 | 0.00 |
|  | Mother's education | 0.16 | 0.47 | 0.08 | 0.30 | 0.00 |
|  | Health insurance | 0.00 | -0.01 | 0.00 | 0.00 | 0.26 |
|  | Residual |  |  |  | -0.07 |  |
| Treatment for diarrhoea | Province | -0.12 | -0.09 | 0.01 | 0.28 | 0.00 |
|  | Urban | 0.02 | 0.43 | 0.01 | 0.25 | 0.17 |
|  | Mother's age | -0.08 | 0.00 | 0.00 | -0.01 | 0.52 |
|  | Wealth index | 0.15 | 0.27 | 0.04 | 1.10 | 0.02 |
|  | Mother's education | -0.05 | 0.47 | -0.02 | -0.58 | 0.01 |
|  | Health insurance | 0.00 | -0.01 | 0.00 | 0.00 | 0.35 |
|  | Residual |  |  |  | -0.04 |  |
| Treatment for ARI | Province | -0.09 | -0.09 | 0.01 | 0.29 | 0.00 |
|  | Urban | 0.02 | 0.43 | 0.01 | 0.24 | 0.06 |
|  | Mother's age | -0.05 | 0.00 | 0.00 | -0.01 | 0.40 |
|  | Wealth index | 0.06 | 0.27 | 0.02 | 0.56 | 0.06 |
|  | Mother's education | -0.01 | 0.47 | 0.00 | -0.13 | 0.37 |
|  | Health insurance | 0.00 | -0.01 | 0.00 | 0.00 | 0.44 |
|  | Residual |  |  |  | 0.05 |  |

# Appendix Table 7. Odds Ratios from Logistics Regression on CHE (By Year)

|  | 2007 | | 2013 | | 2018 | |
| --- | --- | --- | --- | --- | --- | --- |
|  | CHE at 10% | CHE at 25% | CHE at 10% | CHE at 25% | CHE at 10% | CHE at 25% |
| Wealth index quintile (1 as reference group) |  |  |  |  |  |  |
| 2 | 0.761*** | 1.254 | 0.843* | 0.719 | 0.716*** | 0.763 |
|  | (0.0741) | (0.303) | (0.0739) | (0.156) | (0.0510) | (0.152) |
| 3 | 0.572*** | 0.938 | 0.646*** | 0.648* | 0.705*** | 0.682* |
|  | (0.0595) | (0.235) | (0.0626) | (0.148) | (0.0534) | (0.145) |
| 4 | 0.454*** | 0.925 | 0.535*** | 0.556** | 0.510*** | 0.384*** |
|  | (0.0590) | (0.309) | (0.0643) | (0.148) | (0.0489) | (0.103) |
| 5 (most wealthy) | 0.323*** | 0.471* | 0.390*** | 0.448** | 0.420*** | 0.420*** |
|  | (0.0548) | (0.211) | (0.0579) | (0.155) | (0.0494) | (0.140) |
| Household head characteristics |  |  |  |  |  |  |
| employed | 0.751** | 0.743 | 0.863 | 0.557** | 0.704*** | 0.494*** |
|  | (0.0837) | (0.196) | (0.0876) | (0.129) | (0.0547) | (0.103) |
| percent of household members older than 65 | 1.847** | 2.440 | 2.217*** | 2.785* | 3.250*** | 2.768** |
|  | (0.544) | (1.673) | (0.555) | (1.648) | (0.637) | (1.300) |
| percent of household members younger than 5 | 0.964 | 1.116 | 1.583** | 1.022 | 2.450*** | 1.892 |
|  | (0.245) | (0.691) | (0.325) | (0.483) | (0.410) | (0.965) |
| N | 15,356 | 15,356 | 17,782 | 17,782 | 24,631 | 24,631 |
| Standard error is reported in parentheses. * p<0.1, ** p<0.05, *** p<0.01 | | |  |  |  |  |

# Appendix Table 8. Coefficients from Sartori's Two-Step Model (By Year)

|  | 2007 | | 2013 | | 2018 | |
| --- | --- | --- | --- | --- | --- | --- |
|  | CHE at 10% | CHE at 25% | CHE at 10% | CHE at 25% | CHE at 10% | CHE at 25% |
| Panel A: Selection |  |  |  |  |  |  |
| Wealth index quintile (1 as reference group) |  |  |  |  |  |  |
| 2 | -0.177 | -0.177 | 0.0417 | 0.0413 | -0.0598 | -0.0611 |
|  | (0.160) | (0.160) | (0.171) | (0.171) | (0.137) | (0.137) |
| 3 | -0.289* | -0.284* | 0.0432 | 0.0389 | 0.0293 | 0.0333 |
|  | (0.153) | (0.153) | (0.170) | (0.170) | (0.147) | (0.146) |
| 4 | -0.467*** | -0.463*** | -0.0398 | -0.0387 | -0.0329 | -0.0291 |
|  | (0.155) | (0.155) | (0.178) | (0.177) | (0.152) | (0.151) |
| 5 (most wealthy) | -0.415** | -0.414** | -0.211 | -0.209 | -0.167 | -0.163 |
|  | (0.167) | (0.167) | (0.189) | (0.188) | (0.162) | (0.162) |
| Household head characteristics |  |  |  |  |  |  |
| employed | -0.0813 | -0.0942 | -0.00274 | -0.00243 | 0.262** | 0.263** |
|  | (0.0963) | (0.0959) | (0.129) | (0.129) | (0.111) | (0.110) |
| percent of household members older than 65 | -0.271 | -0.300 | -0.817*** | -0.849*** | -0.579** | -0.601** |
|  | (0.241) | (0.243) | (0.279) | (0.280) | (0.251) | (0.252) |
| percent of household members younger than 5 | 0.0924 | 0.0801 | 0.436 | 0.439 | 0.492* | 0.485* |
|  | (0.225) | (0.225) | (0.328) | (0.327) | (0.279) | (0.279) |
| Panel B: Outcome |  |  |  |  |  |  |
| Wealth index quintile (1 as reference group) |  |  |  |  |  |  |
| 2 | -0.159*** | 0.0879 | -0.0744* | -0.114 | -0.160*** | -0.0707 |
|  | (0.0454) | (0.0894) | (0.0409) | (0.0789) | (0.0350) | (0.0752) |
| 3 | -0.311*** | 0.00192 | -0.211*** | -0.157* | -0.184*** | -0.139* |
|  | (0.0492) | (0.0954) | (0.0446) | (0.0845) | (0.0374) | (0.0808) |
| 4 | -0.423*** | -0.0660 | -0.329*** | -0.219** | -0.359*** | -0.330*** |
|  | (0.0567) | (0.111) | (0.0532) | (0.102) | (0.0450) | (0.100) |
| 5 (most wealthy) | -0.576*** | -0.200 | -0.412*** | -0.290** | -0.489*** | -0.427*** |
|  | (0.0715) | (0.142) | (0.0656) | (0.127) | (0.0553) | (0.120) |
| Household head characteristics |  |  |  |  |  |  |
| employed | -0.146*** | -0.0843 | -0.0596 | -0.256*** | -0.179*** | -0.270*** |
|  | (0.0472) | (0.0890) | (0.0447) | (0.0819) | (0.0369) | (0.0706) |
| percent of household members older than 65 | 0.636*** | 0.522** | 0.538*** | 0.576*** | 0.739*** | 0.569*** |
|  | (0.139) | (0.238) | (0.126) | (0.209) | (0.103) | (0.177) |
| percent of household members younger than 5 | -0.00475 | 0.0552 | 0.299*** | -0.00661 | 0.476*** | 0.314* |
|  | (0.107) | (0.204) | (0.0944) | (0.187) | (0.0802) | (0.179) |
| N | 15,356 | 15,356 | 17,784 | 17,784 | 24,632 | 24,632 |
| Standard error is reported in parentheses. * p<0.1, ** p<0.05, *** p<0.01 | | |  |  |  |  |

Appendix Figure 1. Percentage of Children without Any Vaccination by Wealth Index in 2018


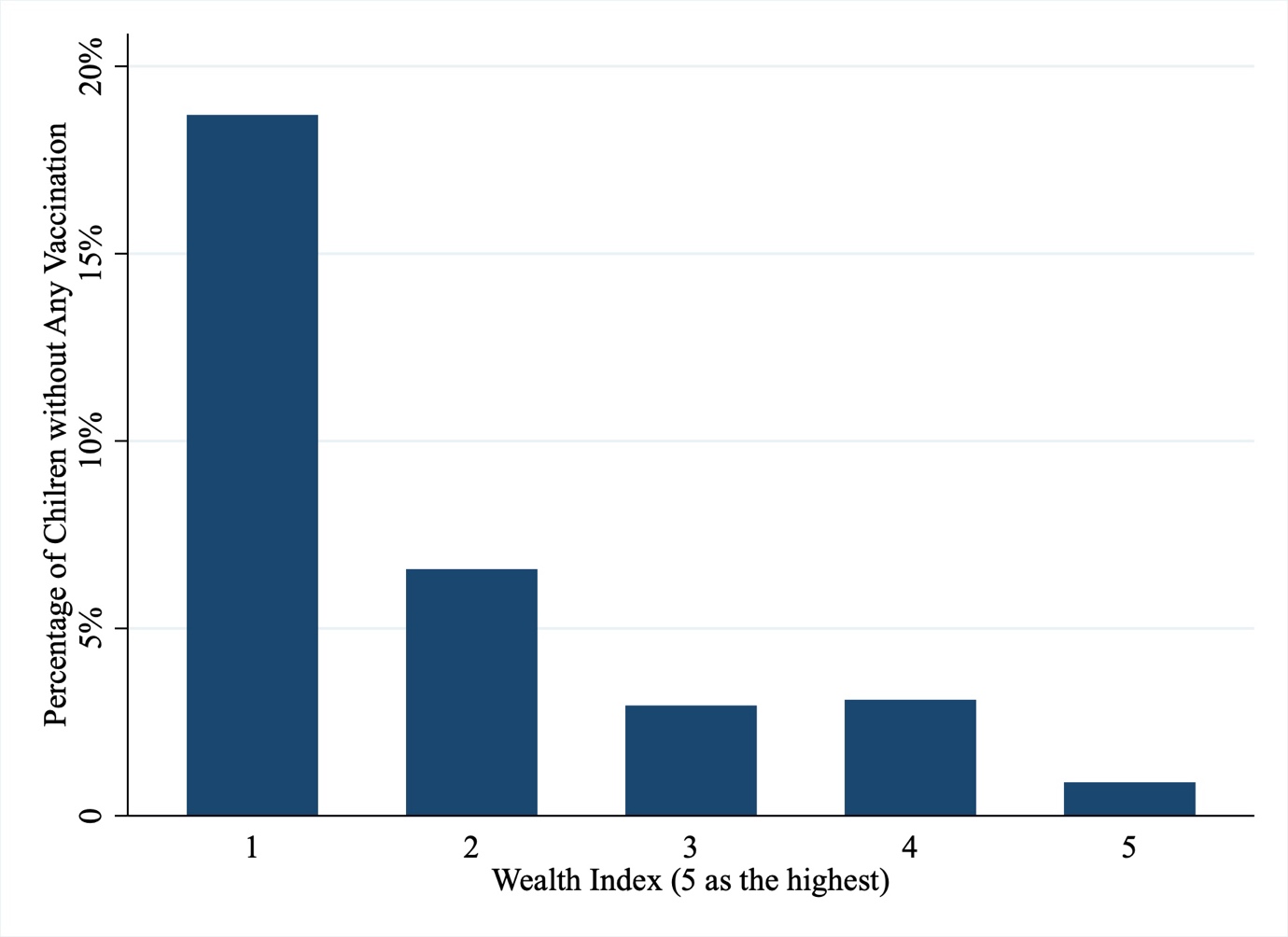


Appendix Figure 2. Percentage of Not-Fully-Vaccinated Children and Percentage of Children without Any Vaccination in the Lowest Wealth Index Quintile in 2018


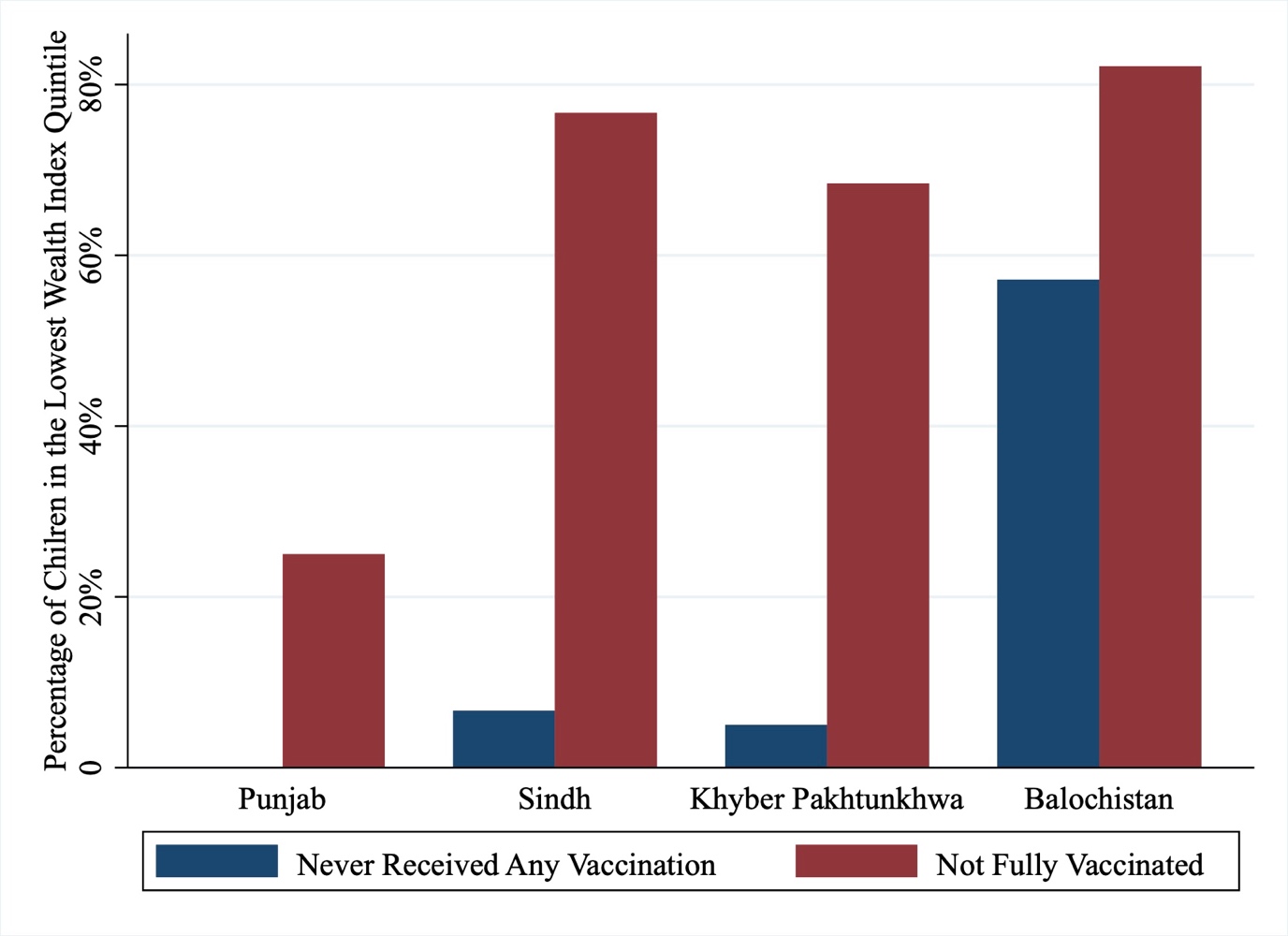

Supplement: Supplementary file 2 — Supplementary Material 2 [file 12939_2024_2232_MOESM2_ESM.docx]
